# Gastroretentive raft liquid delivery system as a new approach to release extension for carrier-mediated drug

**DOI:** 10.1080/10717544.2018.1474969

**Published:** 2018-05-24

**Authors:** Samar M. Abouelatta, Ahmed A. Aboelwafa, Omaima N. El-Gazayerly

**Affiliations:** a Department of Pharmaceutics, Faculty of Pharmacy, Ahram Canadian University, Cairo, Egypt;; b Department of Pharmaceutics, Faculty of Pharmacy, Cairo University, Cairo, Egypt

**Keywords:** Gabapentin, raft forming systems, GMO, LM-pectin 101, gellan gum

## Abstract

Gabapentin (GBP), an antiepileptic and anti-neuropathic agent, suffers from short half-life (5–7 h), has narrow absorption window, and is absorbed via carrier-mediated mechanism resulting in frequent dosing, poor compliance, and poor bioavailability (<60%). Moreover, GBP is a freely water-soluble drug, thus it is considered a challenging candidate to be formulated as extended release dosage form. In this study, raft forming systems were investigated as a potential drug delivery system for prolonging gastric residence time of GBP. A 2^3^ full factorial design was adopted to study the effect of formulation variables (% gellan gum, % GMO, and % LM-pectin 101), on the percent of GBP released at different time intervals (1, 5, and 8 h) as well as the gel strength, and thus was achieved an optimized formula with zero-order release profile suitable for once-daily administration. *In vivo* assessment was performed in rats to evaluate gastric residence of the gel formed. In addition, the oral bioavailability of GBP relative to commercially available Neurontin^®^ immediate release oral solution was also investigated. Significant increase was observed for *C*_max_, AUC_(0–_*_t_*_)_, and AUC_(0–∞)_. The increase in relative bioavailability of GBP from the optimized formula was 1.7 folds.

## Introduction

Gabapentin (GBP) is an anticonvulsant that was initially developed and approved as an adjunctive therapy for the treatment of partial seizures in both the adult and pediatric populations (Beal et al., 2012). GBP has been approved by the US Food and Drug Administration (FDA) for the treatment of post-therapeutic neuralgia (PHN; Kong & Irwin, 2007; Gordi et al., 2008). GBP is absorbed in the upper gastrointestinal tract via a saturable L-amino acid transport system which is partially responsible for the nonlinear absorption of GBP and the inverse correlation between bioavailability and dose (McLean, 1994). In addition, it has a short half-life (5–7 h) requiring a dosing regimen of at least three times daily, resulting in demonstrable fluctuations in plasma levels throughout the day (Gordi et al., 2008).

In 2011, a gastroretentive (GR) formulation of GBP tablets (G-GR, Gralise^®^) was approved by the FDA for the treatment of PHN. The effective dosing regimen of G-GR is 1800 mg, once daily taken with the evening meal (Chen et al., 2013b). GBP extended-release with AcuForm technology increased the bioavailability of GBP via two separate mechanisms; it released the medication at a slower rate, which prevented saturation of the active transport mechanism; and the AcuForm technology allowed retention in the stomach for up to 8 h, enabling delivery of GBP to the optimal site of absorption for approximately 10 h (Gordi et al., 2008; Beal et al., 2012).

Gralise^®^ is only commercially available as tablets containing 300 mg or 600 mg of GBP; the tablet size was found to be approximately 10 mm and was labeled to be swallowed as a whole without crushing, splitting, or even chewing (Chen et al., 2013a).

Special populations (elderly, pediatrics) who suffer from swallowing dysfunction (Kelly & Wright, 2009; Shimoyama et al., 2012) face great challenge not being able to take this controlled release medication resulting in patient noncompliance. Conventional liquid formulations (Neurontin^®^ oral solution) are commercially available but without the required extended release properties. Formulating GBP as extended release liquid oral delivery system would be of great advantage for this kind of special population.

The floating raft system (FRS) is an advanced revolution in liquid oral controlled drug delivery. These systems are liquids at room temperature but undergo gelation when come in contact with body fluids or change in pH. They have a unique property of temperature dependent and cation-induced gelation, which has been assessed for sustaining drug delivery and targeting (Prajapati et al., 2013; Abou Youssef et al., 2015). The formed gels remain intact within the stomach contents for several hours so increased gastric residence time resulting in prolonged drug delivery in the gastrointestinal tract (Kerdsakundee et al., 2015). Raft systems could offer a revolutionary solution for formulation liquid oral extended release dosage form for drugs whose absorption is carrier mediated as GBP.

To overcome the increasing costs and time consumption of experimentation during the development of novel drug delivery systems, dry lab term is now predominating. This is achieved through developing and optimizing the formulation with few experiments. Optimization of formulations using statistical experimental designs is a powerful and efficient tool in the development of pharmaceutical dosage forms (Lewis et al., 1998).

In this study, the main aim is to improve the bioavailability of GBP via formulating a statistically optimized FRS characterized by controlled release for a prolonged period of time with maximum gel strength. A 2^3^ full factorial design was tailored to screen the influence of the studied factors namely: % gellan gum, % Glyceryl monooleate (GMO), and % LM-pectin 101) on the percent of GBP released at different time intervals (1, 5, and 8 h) as well as the gel strength. Desirability function was then used to get an optimized formula. The pharmacokinetic properties of the optimized GBP raft forming systems was then compared to the marketed product Neurontin^®^ oral solution (250 mg/5 ml).

## Materials and methods

### Materials

GBP was supplied as a kind gift sample from Al Andalous Pharmaceuticals (Giza, Egypt). Eudragit NE 30D was obtained as a free generous gift from Evonik (GmbH, Darmstadt, Germany). Kelcogel CG-LA (Gellan gum) was supplied as a generous sample gift from CP kelco (Lille Skensved, Denmark). LM-101 pectin with DE of 36% Kelco (Lille Skensved, Denmark) and was referred as GP36. Glyceryl monooleate (GMO; HLB = 3) was obtained as gift sample from M/s Gattefosse (St Priest, Cedex, France). Calcium chloride dihydrate, sodium citrate dihydrate, potassium dihydrogen orthophosphate, and Tween 80 were obtained from El-Nasr Company for Chemicals and Pharmaceuticals. Hydrochloric acid (HCl) was of analytical reagent grade and acetonitrile (HPLC) was purchased from Sigma Chemical Co. (St. Louis, MO, USA).

### Formulation of GBP raft forming dosage form using 2^3^ full factorial design

#### Experimental design

Factorial designs are now considered as role model in pharmaceutical applications for studying the influence of independent variables (Factors) on their corresponding responses. The process of optimization is achieved through an empirical model equation for the response as a function of the different variables (Abouelatta et al., 2015).

In the present study, 2^3^ full factorial design was adopted to study the effect of different variables on their corresponding responses using Design-Expert^®^ software (version 7; Stat-Ease, Inc., Minneapolis, MN). The studied factors were: A: % Gellan gum, B: % GMO, and C: % of Pectin LM-101, each at two levels as shown in Table 1.

**Table 1.** 2^3^ full factorial design layout for formulation of GBP rafts: factors and responses.

	Level used	
Factors (independent variables)	−1	+1
A: % Gellan gum	0.5	1.5
B: % GMO	20	30
C: % LM-pectin	0	1
Responses (dependent variables)	Constraints	
Y_1_: Release (%) after 1 h	8% ≤ Y_1_ ≤ 12%, target = 10%	
Y_2_: Release (%) after 5 h	30% ≤ Y_2_ ≤ 40%, target = 35%	
Y_3_: Release (%) after 8 h	50% ≤ Y_3_ ≤ 60%, target = 55%	
Y4: Gel strength	Maximize to 3	

In order to minimize the error in the 2^3^ full factorial design duplicate experimentation were carried out. Sixteen formulae were suggested and randomly arranged by Design Expert^®^ 7 software. The percent of drug released after 1, 5, and 8 h in addition to gel strength were selected as dependent variables.

### Preparation of GBP floating gastroretentive raft forming formulae

#### Coating of drug with Eudragit NE 30D

GBP was first dispersed in Eudragit NE 30D and then dried in an oven at 70 °C till completely dry. The achieved mixture was then kept in tightly closed containers until further used in the preparation of raft forming sols.

#### Preparation of floating GBP sols

The 16 suggested formulae were prepared according to Itoh et al. (2007) briefly, both gellan gum and pectin LM-101 were added to deionized water containing 0.17% (w/v) of sodium citrate and 0.045% (w/v) calcium chloride while heating to 40–50 °C and stirring on a magnetic stirrer (model SP 72220-26, Barnstead Thermolyne, Ramsey, MN, USA). The solution was allowed to cool to 40 °C and GMO was then added. The previously coated drug was suspended in the resulting solution and finally stored in amber color bottles until further use (Itoh et al., 2007).

### *In vitro* drug release

The release of GBP from floating raft formulae was carried out in USP type (II) dissolution apparatus (Copley, USA) at 50 rpm in 900 ml 0.1-N HCl. The release media was maintained at 37 °C ± 0.1 °C. Ten milliliters of the formulation was carefully added using a disposable syringe to the dissolution vessel without much disturbance (Abou Youssef et al., 2015). At predetermined time intervals, 5-ml aliquots were removed and replaced by fresh media. GBP was further analyzed using a validated HPLC method (Gupta et al., 2008). Tests were carried in duplicates.

### *In vitro* gelling capacity and strength

Two milliliters of the formulation was carefully placed into a measuring cylinder using a pipette and 6 ml of the 0.1 N HCl (pH = 1.2), as gelation solution was added slowly and the gelation was detected by visual examination (Abou Youssef et al., 2015). Strength of the formed gels was examined by pressing the formed gel with a pair of fine forceps. The gelling capacity was scored in three categories based on gelation time and period for which the formed gel remained:

1. Gels after few minutes, dispersed rapidly
2. Gelation immediate remains for 12 h
3. Gelation immediate remains for more than 12 h

### *In vitro* buoyancy of *in situ* formed gel

*In vitro* buoyancy lag time and duration were determined in a USP dissolution apparatus-II with 900 ml 0.1-N HCl (pH = 1.2) at 37 ± 0.1 °C with a paddle speed of 50 rpm. A measured sample of 10 mL was placed in the release media. The time taken for the onset and duration of floating were noted (Rajinikanth & Mishra, 2008). Light photos were used to visualize the floating behavior GBP formed raft.

### Analysis of the factorial design

Collection of mathematical and statistical data is always useful in analyzing the problems in which a response of interest is influenced by several variables and the objective is to optimize this response.

Four responses were studied: the percent of drug released after 1 h (Y_1_), 5 h (Y_2_), 8 h (Y_3_), and gel strength (Y_4_).

The multiple regression equation was carried out by Design Expert^®^ 7 software. The effects of independent variables upon the responses were modeled using the following polynomial equation:

$$\text{Y}={\text{b}}_{0}+{\text{b}}_{\text{1}}\text{A}+{\text{b}}_{\text{2}}\text{B}+{\text{b}}_{\text{3}}\text{C}+{\text{b}}_{\text{12}}\text{AB}+{\text{b}}_{\text{13}}\text{AC}+{\text{b}}_{\text{23}}\text{BC}+{\text{b}}_{\text{123}}\text{ABC}$$

where, Y is the dependent variable, b_0_ is the arithmetic mean response of the 16 runs, and b_1_, b_2_, and b_3_ are the estimated coefficients for the factors A, B, and C, respectively. The main effects (A, B, and C) represent the average result of changing one factor at a time from its low to high values. The interaction terms (AB, AC, BC, and ABC) show how the response changes when two or more factors are simultaneously changed.

### Measurement of viscosity

Viscosity determinations of the prepared FRSs were carried out on a rotating viscometer (DV-II Programmable Rheometer, Brookfield Engineering LABS, Stoughton, MA) using the spindle CPE-41 at 25 ± 2 °C. Viscosity was measured over the range of speed settings from 0.3 to 60 rpm with 20 s between each two successive speeds. Viscosity measurement for each sample was done in duplicate (Tayel et al., 2013; Morsi et al., 2014). The flow behavior was studied according to Farrow’s equation: Log D = N Log S-Log η, where, D is the shear rate (sec-1), S is the shear stress (Pa), N is Farrow’s constant, and η is the viscosity (cp). The flow index (N), characteristic for each formulation, was obtained, where *N* = 1 indicates Newtonian behavior, while N is less than one, it indicates shear rate thickening. If N is greater than one, it indicates shear rate thinning (Rawlins, 1977; El-Nabarawi et al., 2013).

### Evaluation of the optimized GBP raft formula

#### Differential scanning calorimetry

The differential scanning calorimetry (DSC) analyses of pure GBP, gellan gum, pectin LM-101, sodium citrate, calcium chloride, coated drug, physical mixtures of the drug, and excipients (gellan gum, pectin LM-101, sodium citrate, calcium chloride) in the ratio of 1:1 and the formulation prepared were carried out (Schimadzu, Japan) to evaluate any possible drug–excipient interaction. Samples of 2–6 mg were placed in aluminum pans (Al-Crucibles, 40 Al) and sealed. The probes were heated from 25 °C to 200 °C at a rate of 10 °C/min under nitrogen atmosphere.

#### Powder X-ray diffraction (XRD) analysis

The X-ray diffraction study was carried out to characterize the physical form of GBP in samples of optimized formula. The physical state of GBP in the various preparations were recorded at room temperature on X-ray diffractometer (Philips, USA) with mono-chromator Cu radiation (1.542 Ǻ), at 40 kV, 35 mA. The diffractometer was equipped with a 2θ compensating slit, and was calibrated for accuracy of peak positions with silicon pellet. Samples were subjected to X-ray powder diffraction analysis in continuous mode with a step size of 0.02° and step time of 1 s over an angular range of 2–60°2*θ*.

#### Scanning electron microscope (SEM)

Pure GBP crystals and GBP coated with Eudragit NE 30D were investigated using scanning electron microscope to visualize its surface morphology at different magnification powers. The sample for SEM (Jeol JXA-840A, Japan) was prepared by sticking the samples on a double adhesive tape, which stuck to an aluminum stub. The stubs were then coated with gold to a thickness of about 300 Å using Edwards sputter coater. These samples were scanned and photomicrographs were taken.

### *In vivo* study

#### In situ gel-forming property in rat stomach

The studies were reviewed, approved (PI 814), and conducted in accordance with the guidelines of the Research Ethics Committee (REC-FOPCU) as well as the EU Directive 2010/63/EU for animal experiments.

The optimized GBP raft formula was prepared using 0.2% brilliant blue as a dye instead of the drug. One milliliter of the colored formula was orally given to 16 male rats weighing (200–300 g) and fasted for 24 h with free access to water. The stomach was removed at predetermined time intervals (Control, 1, 2, 3, 4, 5, 6, and 8 h). Light photos were taken to prove the formation of gel and its gastric retention for extended period of time (Itoh et al., 2008).

### Experimental design and sample collection

The study was carried out to compare the pharmacokinetic parameters of GBP from the optimized raft forming systems containing 250 mg/5 ml (test product) and the commercially available, immediate release Neurontin^®^ oral solution containing 250 mg/5 ml (reference product), after single oral administration. Two treatments, two periods, randomized, and cross over design was carried out. The protocol and informed consent form were approved by the Faculty of Pharmacy, Cairo University Research Committee (PI 814). Six male healthy human volunteers participated in the study. The study was fully explained to the volunteers before starting the study, and the informed consent forms were signed by each volunteer as per declarations of Helsinki (Brazil 2013).

The volunteers were randomly assigned to one of two groups of equal size. The two-period study was carried out as follows: Period I, three human volunteers (group1) received Neurontin^®^ oral solution and the other three human volunteers (group 2) received optimized GBP raft forming system, equivalent to 250 mg/5 ml GBP. The volunteers were asked to fast for at least 10 h with free water access, till 1 h before treatments administration. Treatments were given with about 240 ml water. They were only allowed to take water after 2 h and a standard meal was given after 4 h from treatments administration. A 1-week washout period separated the two periods. In period II, the opposite treatment was administered.

The study was supervised by a physician who was also responsible for both the safety of the volunteers and samples collection. Venous blood (5 ml) for assay of plasma concentrations of GBP were obtained via the indwelling cannula immediately prior to dosing and at the predetermined time intervals of 0, 0.5, 1.0, 1.5, 2.0, 2.5, 3.0, 3.5, 4.0, 6.0, 8.0, 10.0, 12.0, and 24 h after oral administration. Samples were collected into polypropylene heparinized tubes and were immediately centrifuged at 3000 rpm for 5 min. The plasma obtained was frozen at −20 °C in labeled tubes till further analysis.

#### Sample preparation

About 50 µl Sildenafil (IS; from a stock solution of concentration 1000 ng/ml) and 600 µl Methanol were added to each sample (450 µl plasma), vortexed for 2 min, and centrifuged for 5 min at 5000 rpm (cooling centrifuge, TGL-20 MB). The supernatant was transferred to another vials filtered through 0.22-μm Millipore filter, then evaporated to dryness using vacuum concentrator (Eppendorf Vacufuge plus, Germany). Dry residues were reconstituted with 200 μl mobile phase (0.5% acetic acid: Acetonitrile [10:90] (v/v)), and finally 50 μl was injected on the column for analysis.

#### LC-MS/MS assay of GBP

Plasma concentrations of GBP were analyzed using a validated LC-MS/MS method. LC system (Shimadzu^®^, Japan) coupled with triple quadrupole MS/MS detector (API 4000^®^, Canada) was used. The chromatographic separation was carried out on Gemini C18 column (2.0 × 150 mm i.d., 5 μm; Phonomenex Inc. Torrance, CA, USA). The mobile phase consisted of 0.5% acetic acid:acetonitrile [10:90] (v/v).The flow rate was set as 0.5 ml/min. The analysis was operated at the MRM (multiple reaction monitoring) mode, and its MS parameters are shown in Table 2 (Park et al., 2007).

**Table 2.** Multiple reaction monitoring (MRM) conditions.

Compound name	Precursor ion (Da)	Product ion (Da)	Dwell time (ms)	Collision energy (v)	Polarity
Sildenafil (IS)	474.5	311.4	800	41.8	Positive
Gabapentin	172.8	155.3	800	16.4	Positive

#### Pharmacokinetic study and statistical analysis

The pharmacokinetic parameters were determined after oral administration of the two treatments of GBP, from plasma concentration time profiles by means of non-compartmental analysis using Kinetica^®^ (version 5, Thermo Fischer Scientific). Maximum GBP concentration (*C*_max_, µg/ml) and time required to reach maximum GBP concentration (*T*_max_, h.) were obtained from the individual plasma concentration time curves. The elimination rate constant (K, h^−1^) was obtained from the slope of the linear regression of the log-transformed plasma concentration–time data in the elimination phase. The area under the plasma concentration–time curve (AUC, µg. h/ml) was subdivided into AUC_(0–24)_ (was determined as the area under the plasma concentration–time curve up to the last measured sampling time and calculated by the linear trapezoidal rule.), and area under the curve from zero to infinity (AUC_(0–∞)_, µg. h/ml), where AUC_0–∞_ = AUC_0–24_ + C*_t_*/K (C*_t_* is the last measured sample concentration at time *t*).

Different pharmacokinetic parameters, *C*_max_, AUC_(0–24)_, and AUC_(0–∞),_ were compared between treatments (Neurontin^®^ oral solution 250 mg/5 ml and GBP raft forming systems) with ANOVA test for the untransformed data. A *p* value of <.05 was considered statistically significant.

## Results and discussion

### *In vitro* buoyancy of *in situ* formed gel

**Figure 1.**
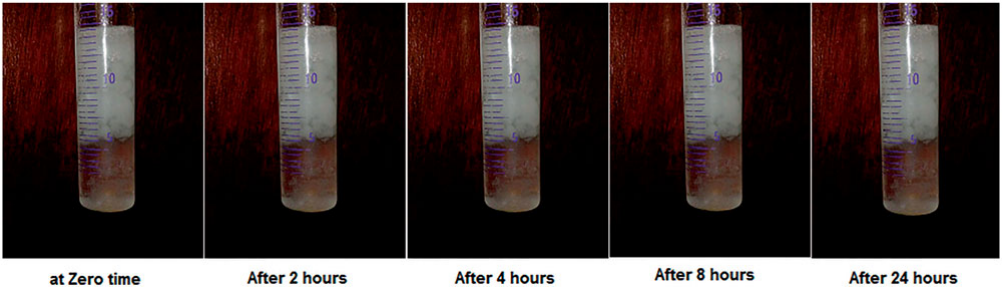
Light photos showing floating behavior of optimized GBP raft formula at different time intervals in 0.1 N HCl.

The formed *in situ* gel floated immediately with no lag time as shown in the attached video. This might be attributed to the presence of high percent of GMO in the formulation. GMO is known to form low dense mass upon contact with 0.1 N HCl due to its oily nature. The formed *in situ* gel remained floating for more than 24 h, and this encourages further *in vivo* study. Figure 1 shows the floating behavior of the optimized GBP raft formula at different time intervals.

### Analysis of the factorial design

**Figure 2.**
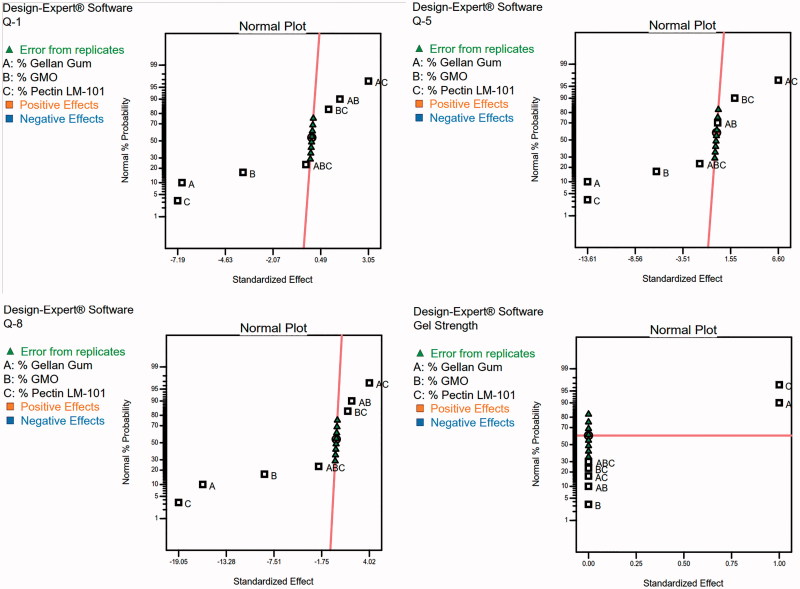
Normal plot of GBP raft formulae for screening of the influence of the studied factors.

To design a new formulation, it is of paramount importance to identify the influencing parameters, since these will affect the properties of the final dosage form. The experimental design method analyzes the influence of different variables on the properties of the drug delivery system (Fernandes et al., 2017). A 2^3^ full factorial design was used to describe the relation between the response under question and the variables studied. Each response was individually analyzed. The eight experimental runs coupled to their observed responses are shown Table 3. ANOVA test was used to measure the level of significance of the tested factors on % GBP released after 1,5, and 8 h (Y_1_, Y_2_, and Y_3_) and Gel strength (Y_4_) as shown in Table 4 . This helps the process of optimization by providing an empirical model equation for the response as a function of the different variables as represented in Table 5. The normal plot of different studied factors and their influence on the observed responses was illustrated in Figure 2.

**Table 3.** Composition of the prepared GBP formulations in actual values (un-coded units) and their observed responses.

	Factors			Responses^a^			
	A	B	C	Y_1_	Y_2_	Y_3_	Y_4_
1	0.5	0	20	27.94 ± 0.01	67.48 ± 0.01	99.18 ± 0.1	1
2	0.5	0	30	21.47 ± 0.01	57.20 ± 0.06	84.55 ± 0.23	1
3	1.5	0	20	15.59 ± 0.1	45.68 ± 0.49	74.34 ± 0.32	2
4	1.5	0	30	13.25 ± 0.16	38.49 ± 0.18	68.72 ± 0.49	2
5	0.5	1	20	16.24 ± 0.11	43.64 ± 0.46	72.63 ± 0.44	2
6	0.5	1	30	12.44 ± 0.16	40.19 ± 0.9	65.60 ± 0.4	2
7	1.5	1	20	10.99 ± 1.07	37.30 ± 0.13	60.70 ± 0.52	3
8	1.5	1	30	9.44 ± 0.24	32.26 ± 0.03	52.76 ± 0.63	3

a Mean ± SD (*n* = 2).

**Table 4.** Coefficient estimates for different model terms appearing in the final equation for each response and their significance levels.

	Y_1_		Y_2_		Y_3_		Y_4_	
	CE	*p* Value	CE	*p* Value	CE	*p* Value	CE	*p* Value
b_0_	15.88	<.0001*	45.27	<.0001*	72.22	<.0001*	2	<.0001*
b_1_	−3.48	<.0001*	−6.81	<.0001*	−8.06	<.0001*	0.5	<.0001*
b_2_	−1.84	<.0001*	−6.81	<.0001*	−9.53	<.0001*	0.5	<.0001*
b_3_	−3.60	<.0001*	−3.16	<.0001*	−4.33	<.0001*	–	
b_12_	1.53	<.0001*	3.30	.5183	2.01	<.0001*	–	
b_13_	0.76	<.0001*	0.086	<.0001*	0.94	<.0001*	−	
b_23_	0.46	<.0001*	0.99	<.0001*	0.71	.0001*	–	
b_123_	−0.15	.0438*	0.86	.0001*	−1.05	<.0001*	–	

CE: coefficient estimates.

* Significant at *p* < .05.

**Table 5.** Coefficient estimates of the fitted regression model in terms of coded factors for the responses and the corresponding *R*^2^ values.

Response	Equation
Y_1_ (% drug released after 1 h)	Y_1_ = 15.88 − 3.48A − 1.84B − 3.60C + 1.53AB +0.76AC +0.46BC −0.15ABC (*R*^2^ = 0.9958)
Y_2_ (% drug released after 5 h)	Y_2_ = 45.27 − 6.81A − 6.81B − 3.16C + 3.30AB +0.086AC +0.99BC −0.86ABC (*R*^2^ = 0.9955)
Y_3_ (% drug released after 8 h)	Y_3_ = 72.22 − 8.06A − 9.53B − 4.33C + 2.01AB +0.94AC +0.71BC −1.05ABC (*R*^2^ = 0.9981)
Y_4_ (gel strength)	Y_4_ = 2 + 0.5 A + 0.5B (*R*^2^ = 1)

The percent of drug released at different time intervals (Y_1_, Y_2_, and Y_3_) at (1, 5, and 8 h) respectively were antagonistically affected by the three studied factors (A, B, and C).

Drug release study was conducted in acidic medium (0.1 N HCl) since our objective was to formulate a gastroretentive dosage form. The release study was carried out in 0.1 N HCl (pH = 1.2) at 37 °C ± 0.1 °C at 50 rpm (Kerdsakundee et al., 2015).

**Figure 3.**
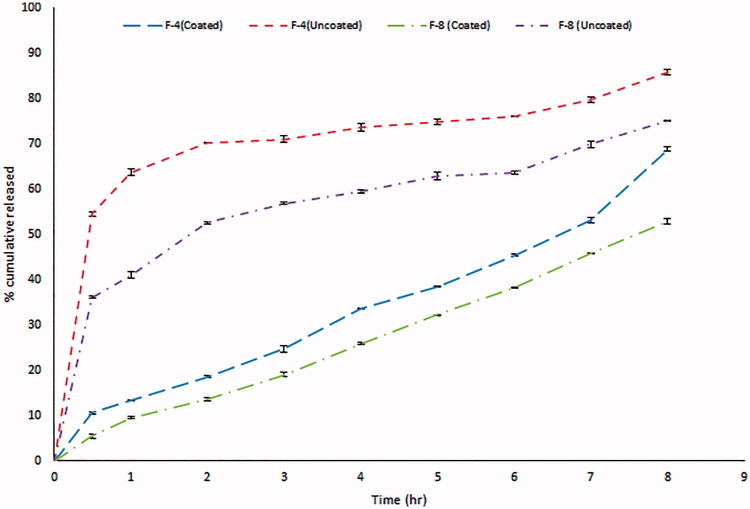
*In vitro* release profile of GBP before and after coating with Eudragit NE 30D.

GBP is freely soluble in water over the whole pH range (Moffat et al., 2004). Figure 3 demonstrates the difference in the release patterns of 2 formulae, 4 and 8 as an example to show the effect of coating of GBP with Eudragit NE 30D. Optimum amounts of sodium citrate and calcium chloride were used to maintain the formulation in the sol state to facilitate the swallowing process but were sufficient to ensure gelation in acidic conditions in the stomach (Rajinikanth & Mishra, 2008). Sodium citrate (0.17% w/v) was added as a chelating agent; it complexes the free Ca^2+^ ions and only releases them in the acidic environment of the stomach (Abou Youssef et al., 2015). Accordingly, the cross-linking of the gellan network and eventually the gelation due to the Ca^++^ ions occurred instantaneously in the stomach. However, a lag time is still evident before the complete gel formation that is why burst release was observed in the first hour (60% release) as shown in Figure 3 (Rajinikanth & Mishra, 2008). We succeeded to overcome this obstacle by application of the following approaches:

Coating GBP with Eudragit NE 30D played a key role in diminishing the percent of GBP released in the first hour, thus a promising controlled release profile of GBP was observed as clearly shown in Figure 3.

Moreover, significant further decrease in the rate and extent of drug release was observed with the increase in the density of the polymer matrix. This might be attributed to the exopolysaccharide anionic nature of gellan gum (Nagarwal et al., 2009). Incorporation of LM-101 pectin as anionic polysaccharide played additional role and resulted in slower release profile for GBP than formulations containing gellan gum only due to formation of firm gel when LM-101 pectin contacts with free Ca^2+^ forming steric entanglements and thus difficulty of GBP release through gels (Itoh et al., 2007).

**Figure 4.**
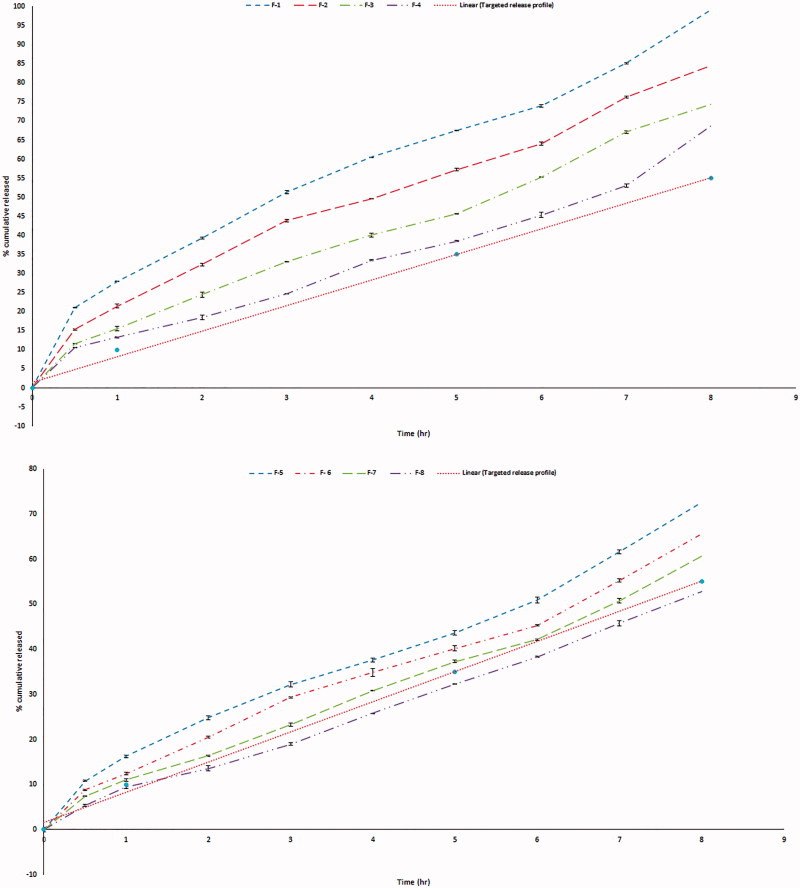
*In vitro* release profile of GBP in 0.1 N HCl (F-1 to F-8).

Finally, the lipophilic nature, low HLB of GMO (Liu et al., 2011) as well as high viscosity of the cubic phase formed upon contact with water, slowed the diffusion of drug molecules through the formed gel (Abou Youssef et al., 2015) resulting in significant retardation of GBP release upon increasing the percent of GMO as shown in Figure 4.

**Figure 5.**
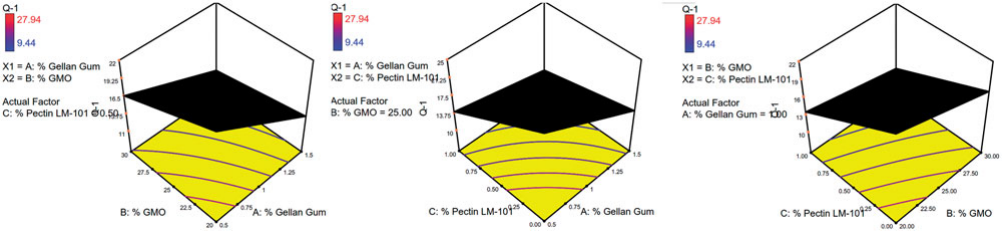
3D surface plot of GBP raft floating formulae after 1 h release in 0.1 N HCl.

**Figure 6.**
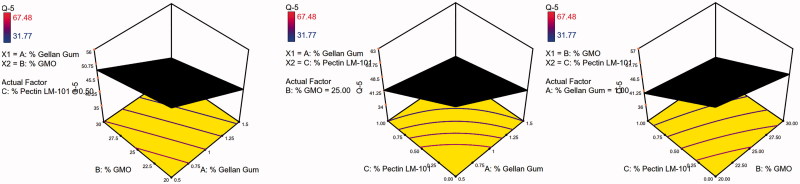
3D surface plot of GBP raft floating formulae after 5 h release in 0.1 N HCl.

**Figure 7.**
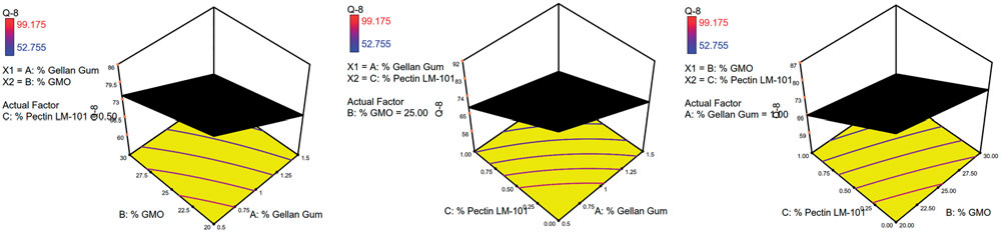
3D surface plot of GBP raft floating formulae after 8 h release in 0.1 N HCl.

The 3D surface plots were further elucidated to study the interaction between the studied factors and their influence on the percent of drug released after 1, 5, and 8 h as shown in Figures 5–7.

Concerning the strength of formed gel (Y_4_), synergistic effect was observed due to increasing chain interaction upon increasing polymer (factors A and C) concentration (Rajinikanth et al., 2007). The contrary, gel strength was reduced upon increasing the semisolid GMO which formed only swollen mass not gel on contacting fluids.

### Optimization of GBP raft formulation

Based on the previous *in vitro* findings, an optimized formula with zero-order release profile of GBP over prolonged time and maximum gel strength was achieved through Design Expert^®^7 software as shown in Table 6.

**Table 6.** The observed and predicted values for the optimized GBP raft formulation.

Factor	Optimized level (D = 0.891)		
A: % Gellan gum	1.42		
B: % GMO	28.49		
C: % Pectin LM-101	1		
Response	Expected	Observed	Residual^a^
Y_1_: Release (%) after 1 h	10.03	10.44	−0.41
Y_2_: Release (%) after 5 h	33.54	38.31	−4.77
Y_3_: Release (%) after 8 h	55	53.94	1.06
Y_4_: Gel strength	2.92	3	−0.8

a Residual = expected value – observed value.

**Figure 8.**
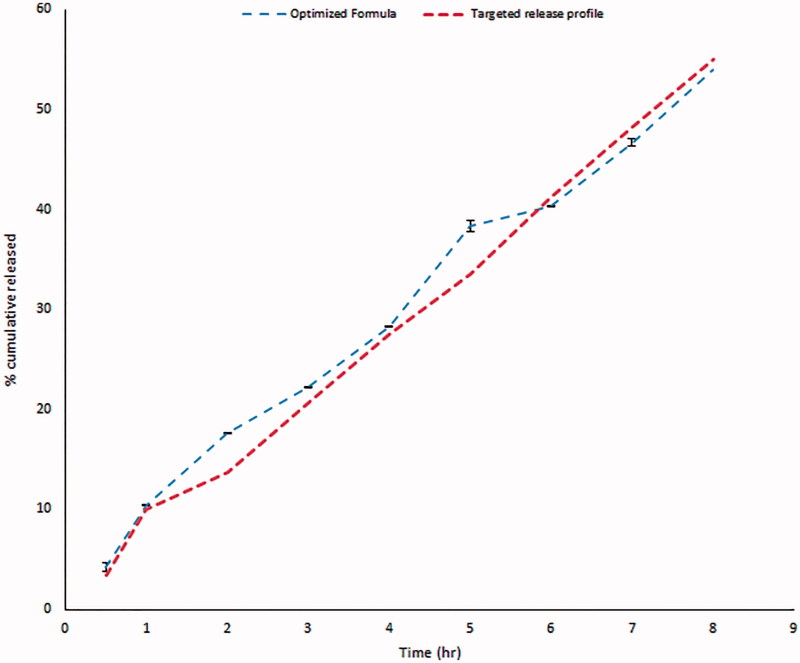
*In vitro* release profile of GBP from the optimized formula in 0.1 N HCl.

Validity of the design was carried out through a check point batch. The batch was prepared according to the predetermined levels given by Design Expert^®^7 software. Both expected and observed release profiles were reported as shown in Figure 8. Minimum residual was calculated thus proofing validity of the design.

### Measurement of viscosity of GBP raft formulations

The formulation should have an optimum viscosity that will allow easy swallowing as a liquid dosage form, which then undergoes a rapid sol–gel transition and floating due to ionic interaction (Rajinikanth & Mishra, 2008).

**Figure 9.**
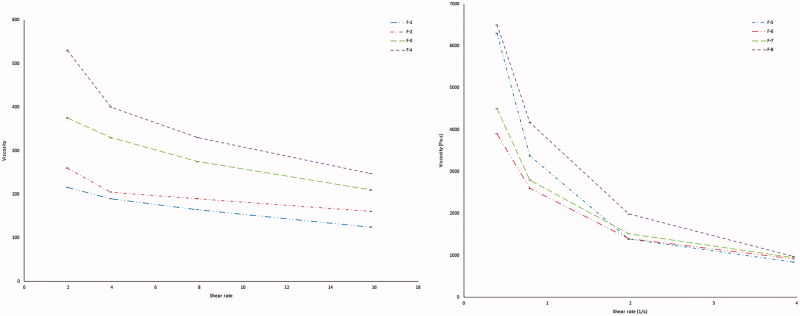
Viscosity of sols of different GBP formulae.

The solutions showed a marked increase in viscosity with increasing concentration of polymers. Both gellan gum and LM-pectin 101 in an ion-free aqueous medium have viscosity close to that of water, and the double helices were only weakly associated with each other by Van der Waals attraction. When gel-promoting cations (Ca^2+^) are present in acidic medium, some of the helices associate into cation-mediated aggregates, which cross-link the polymers (Abou Youssef et al., 2015). Table 7 and Figure 9 show the rheological studies carried for Sols.

**Table 7.** Rheological behavior of different formulae in both sol and gel forms.

Formula code	Viscosity of sols (cps)	*N* (Furrow’s constant of sols)	Flow behavior
F-1	216 ± 0.71	1.502	Shear thinning
F-2	260 ± 0.98	1.29	Shear thinning
F-3	330 ± 1.03	1.35	Shear thinning
F-4	530 ± 2.58	1.45	Shear thinning
F-5	1390 ± 0.09	2.99	Shear thinning
F-6	1400 ± 0.55	3.21	Shear thinning
F-7	1520 ± 1.25	2.54	Shear thinning
F-8	4517 ± 1.66	3.97	Shear thinning
Optimized formula	2360 ± 0.73	2.88	Shear thinning

### Evaluation of the optimized GBP raft formula

#### Differential scanning calorimetry

**Figure 10.**
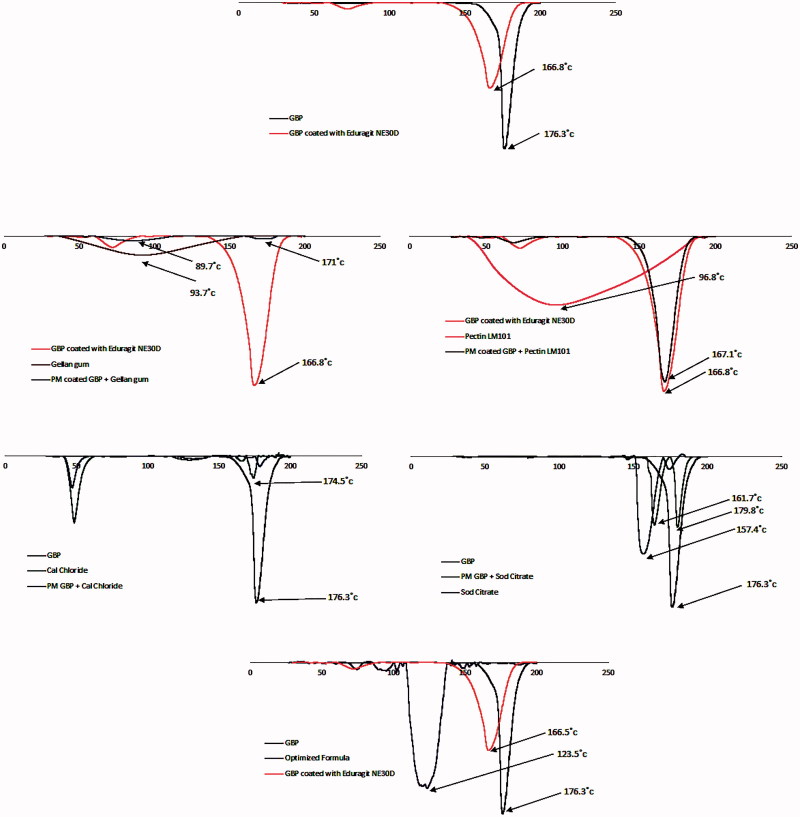
DSC thermograms of GBP, physical mixtures, and optimized formula.

Differential scanning calorimetry (DSC) is a tool used to investigate the crystalline or amorphous nature of the drug within the developed formulations and to elucidate any possible interactions with other ingredients (Basha et al., 2013). As shown in Figure 10, pure GBP showed a sharp endothermic peak at 176.3 °C corresponding to its melting point (Kim et al., 2013), when GBP was coated with Eudragit NE 30D a sharp endothermic peak was observed at 166.8 °C which is also in the reported range of pure GBP.

Gellan gum and LM pectin 101 exhibited a broad endothermic peak at 93.7 °C and 98.6 °C, respectively indicating their melting points (Ahuja et al., 2013; Abouelatta et al., 2016).

Calcium chloride dihydrate showed a sharp endothermic peak at 174.5 °C corresponding to its melting point. In case of sodium citrate dihydrate, single sharp endothermic peak was shown at 157.4 °C indicating its conversion to anhydrous form of sodium citrate.

The sharp endothermic peak of GBP was not changed in physical mixtures of different excipients in the ratio of 1:1 and the drug remained in its intact crystalline form confirming the absence of interaction between the drug and excipients used in formulation.

The optimized formula showed single slightly shifted broad peak at 123.5 °C which might indicate that part of GBP had lost its crystallinity while the other part remained intact during DSC scan.

### Powder X-ray diffraction (XRD) analysis

**Figure 11.**
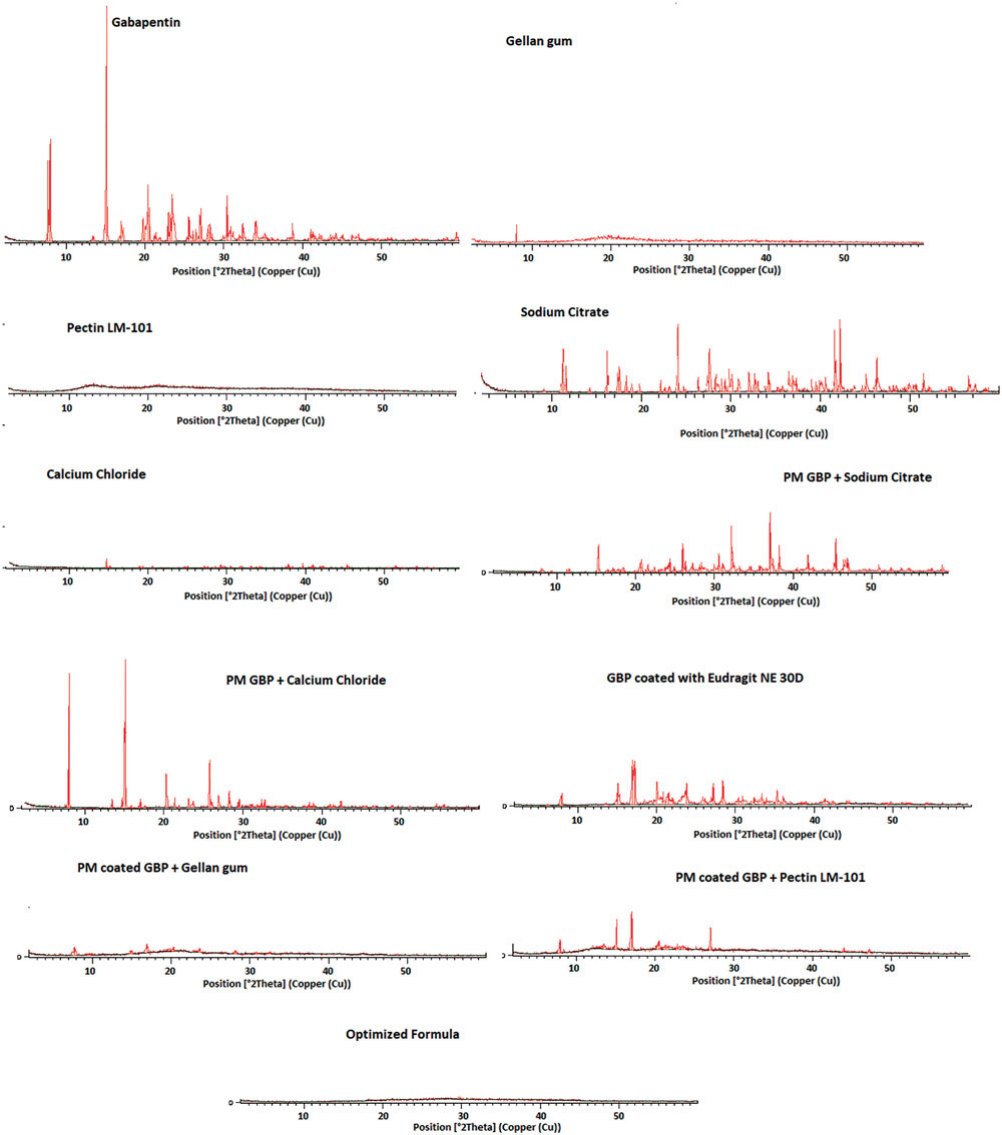
XRD diffractograms of GBP, physical mixtures, and optimized formula.

X-ray diffractograms of pure GBP, GBP coated with Eudragit NE 30D, as well as the used excipients (gellan gum, pectin LM-101, calcium chloride dihydrate, and sodium citrate dihydrate), physical mixtures(1:1), and optimized formula are shown in Figure 11.

GBP showed sharp crystalline peaks at 6.0°, and at 12.1° (Kim et al., 2013). The diffractogram of sodium citrate exhibited sharp peaks at 12°, 25°, and 42°. The diffractograms of gellan gum, pectin LM-101, and calcium chloride dihydrate were typical of amorphous materials with no sharp peaks.

The intensity of GBP peaks was diminished upon coating with Eudragit NE 30D but were kept in the same position.

On studying the PM of coated GBP with both gellan gum and pectin LM-101, no sharp peaks of GBP were observed, this might be due to loss of drug crystallinity during coating process. But GBP maintained its crystallinity in PM with calcium chloride and sodium citrate as the drug was not subjected to coating.

The optimized formula showed no peaks indicating that the drug lost its crystallinity, this might be attributed to both drug coating as well as presence of GMO in the formula which formed cubic form upon contact with water and thus masked the drug crystallinity (Liu et al., 2011).

#### Scanning electron microscope (SEM)

**Figure 12.**
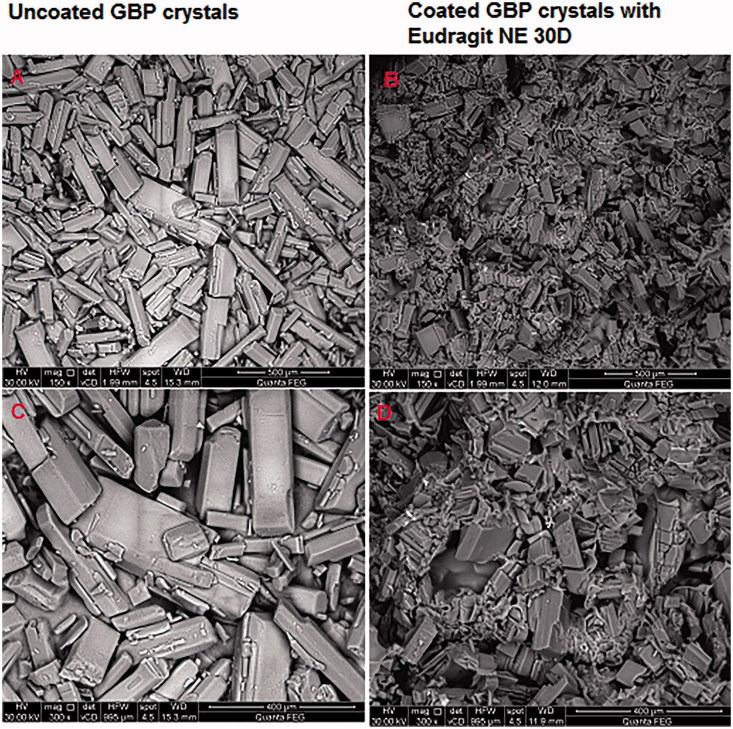
SEM of GBP pure drug crystals and GBP crystals coated with Eudragit NE 30D.

Figure 12 showed SEM micrographs of pure GBP and GBP coated with Eudragit NE 30D at different magnification powers.

Pure GBP was shown as sharp-edged cuboidal crystals. Upon coating GBP with Eudragit NE 30D, these sharp crystals appeared as if they were embedded in Eudragit NE 30D matrix.

#### *In situ* gel forming and gastroretentive property in rat stomach

**Figure 13.**
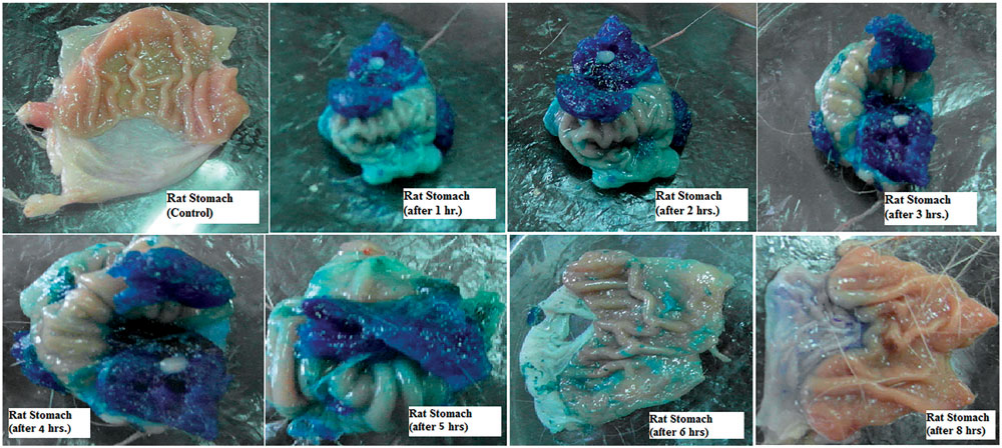
Light photos showing presence of gels in rat stomach at different time intervals after oral administration of optimized formula.

Figure 13 shows the appearance of the gels formed in the rat stomach following oral administration of 1 ml of the dyed optimized formula for 8 h. The presence of blue-colored gel confirms the gastroretentive properties of the drug free optimized formula. The amount of blue gel decreased by time as shown in the figure until completely disappeared after 8 h. The results show slow erosion of the formed gel thus emphasizing its superior gel strength of the optimized formula (Itoh et al., 2008).

#### *In vivo* pharmacokinetic study

**Figure 14.**
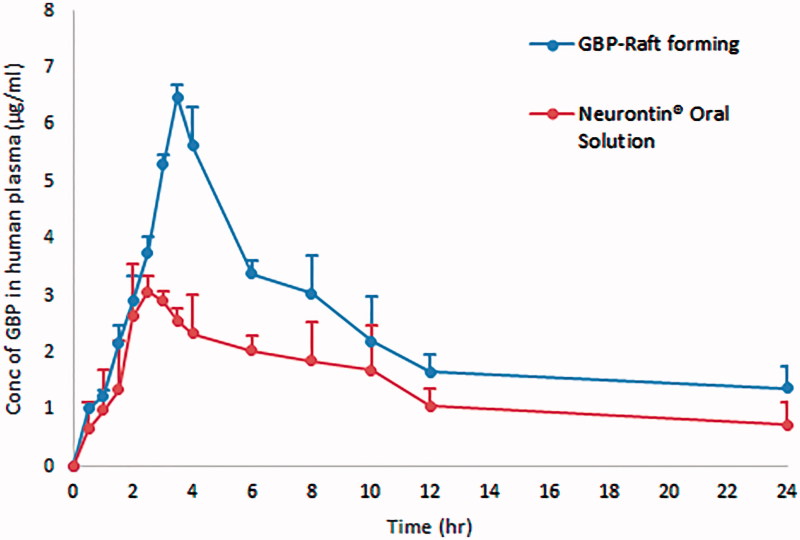
Average plasma concentration time profiles after single oral administration of both GBP raft forming systems and immediate release marketed Neurontin^®^ oral solution to six human volunteers.

Figure 14 represents the average plasma concentration time profiles after single oral administration of both GBP raft forming systems and immediate release marketed Neurontin^®^ oral solution. Different pharmacokinetic parameters were computed by fitting plasma concentration time profiles to non-compartmental analysis as shown in Table 8.

**Table 8.** Average pharmacokinetic parameters of both GBP raft forming systems and immediate release marketed Neurontin^®^ oral solution following the administration to six human volunteers.

Pharmacokinetic parameters	Neurontin^®^ oral solution	Optimized raft forming GBP
*C*_max_ (µg/ml)	3.07 ± 0.83	6.49 ± 0.22*
*T*_max_ (h)	2.5 ± 0.35	3.5
AUC_(0–24)_ (µg h/ml)	32.89 ± 0.86	55.49 ± 6.39*
AUC_(0–∞)_ (µg h/ml)	44 ± 2.36	74.89 ± 4.93*

* Statistically significant (*p* < .05).

Statistically significant difference (*p* < .05) was observed between two treatments for *C*_max_, AUC_(0–24)_, and AUC_(0–∞)_, while statistically insignificant difference (*p* > .05) was obtained for *T*_max_.

Compared to the marketed product, the relative bioavailability was found to be 170% referring to AUC_(0–∞)_. The significant increase in *C*_max_ and higher AUC indicate an increase in the extent of absorption of GBP from the optimized raft forming systems with higher bioavailability and more extended plasma concentration – time profile over 24 h in comparison with the commercially available Neurontin^®^ oral solution. This could be due to the increased gastric residence of GBP at its absorption site.

## Conclusions

In this study, GBP was successfully formulated in an optimized floating raft forming system. GBP was primarily coated by Eudragit NE 30D. The coated drug was further incorporated into the raft forming system. This floating raft forming system is characterized by: having an optimum viscosity that will allow easy swallowing as a liquid dosage form, which then undergoes a rapid sol–gel transition and floating due to ionic interaction. Excellent floating behavior and controlled release profile for more than 12 h were observed. The pharmacokinetic study revealed significant increase in *C*_max_; the relative bioavailability was found to increase by 1.7-fold when compared to immediate release Neurontin^®^ oral solution. Thus, a promising floating raft forming system has been achieved which could be suitable for special populations as geriatrics and pediatrics who find difficulty in swallowing controlled release solid dosage forms of GBP thus leading to improved efficacy due to enhanced patient compliance.
